# Neutrophil Extracellular Traps in Fatal COVID-19-Associated Lung Injury

**DOI:** 10.1155/2021/5566826

**Published:** 2021-07-30

**Authors:** Astrid Obermayer, Lisa-Maria Jakob, Jasmin D. Haslbauer, Matthias S. Matter, Alexandar Tzankov, Walter Stoiber

**Affiliations:** ^1^Department of Biosciences, University of Salzburg, Hellbrunnerstrasse 34, 5020 Salzburg, Austria; ^2^Institute of Pathology and Medical Genetics, University Hospital Basel, Schönbeinstrasse 40, 4031 Basel, Switzerland

## Abstract

An excess formation of neutrophil extracellular traps (NETs), previously shown to be strongly associated with cytokine storm and acute respiratory distress syndrome (ARDS) with prevalent endothelial dysfunction and thrombosis, has been postulated to be a central factor influencing the pathophysiology and clinical presentation of severe COVID-19. A growing number of serological and morphological evidence has added to this assumption, also in regard to potential treatment options. In this study, we used immunohistochemistry and histochemistry to trace NETs and their molecular markers in autopsy lung tissue from seven COVID-19 patients. Quantification of key immunomorphological features enabled comparison with non-COVID-19 diffuse alveolar damage. Our results strengthen and extend recent findings, confirming that NETs are abundantly present in seriously damaged COVID-19 lung tissue, especially in association with microthrombi of the alveolar capillaries. In addition, we provide evidence that low-density neutrophils (LDNs), which are especially prone to NETosis, contribute substantially to COVID-19-associated lung damage in general and vascular blockages in particular.

## 1. Introduction

Since the first published reports on clinical features of COVID-19 (e.g., [1]), there is cumulative evidence that critical cases could be substantially aggravated by the tissue-damaging effects of neutrophil extracellular DNA traps (NETs) [2]. Likely associated with a cytokine storm [3–5], critically ill COVID-19 patients were shown to develop conditions that had all been previously identified as closely associated with NETosis (a topic introduced by Barnes et al. [6] and Mozzini and Girelli [7]) such as severe tissue injury, coagulopathy, and barrier dysfunction of the lungs [8]. A copious release of proinflammatory peptides causing a cytokine storm, as seen in COVID-19 [9], has long been regarded as a potent inductor of NETosis (e.g., [10]).

Present evidence indicates that severe COVID-19 is a specific acute respiratory distress syndrome (ARDS) phenotype frequently showing diffuse alveolar damage (DAD) and presenting with endothelial dysfunction and a hypercoagulable state [11–22]. Previous studies have delineated a central role of NETs in alveolar and vascular damage in ARDS of other etiologies and other inflammatory pulmonary diseases [23, 24] and to intravascular clotting and thrombosis [25–35], indicating a likely pathophysiological association between NETosis and ARDS.

ARDS of various etiologies had previously been shown to be accompanied with elevated serum levels of D-dimers [36], yet considerably less pronounced than in COVID-19, and cell-free DNA [35], both shown to be strongly associated with NETosis (e.g., [37]). A potential link between systemic NETosis and endothelial dysfunction leading to microvascular coagulation in COVID-19 is therefore highly probable. In line with this, COVID-19 sera were found to contain abundant NETosis markers such as cell-free DNA, myeloperoxidase- (MPO-) associated DNA, and citrullinated histone H3 (citH3), together with elevated levels of the acute-phase C-reactive protein (CRP) and D-dimer [38, 39]. Initial immunohistochemical data further substantiated a role of NETosis in COVID-19-associated lung injury and thromboembolic complications [40–42]. Based on this evidence, the potential influential role of NETs and their by-products in COVID-19 pathogenesis and outcome is now rapidly gaining acceptance and has also been considered in treatment approaches in COVID-19 such as anti-IL6 and IL26 therapy (e.g., [5, 43–45]).

This study intends to strengthen and expand upon the above findings. We used immunolabeling together with histochemical staining to trace the NET-forming cells, NETs, and their molecular markers in autopsy lung tissue from seven COVID-19 patients. Stereological point counting was employed to quantify citH3+ NETs and citH3+ neutrophils in these tissues, also to enable comparison with NETosis levels in specimens of non-COVID-19 bacterial pneumonia and DAD, and in healthy control lungs.

## 2. Materials and Methods

All autopsies were performed at the Institute of Pathology, University Hospital of Basel, Switzerland, according to previously delineated safety protocols [18]. COVID-19 lungs were tracheally perfused with 4% phosphate-buffered formalin (72 hrs. at room temperature) and cut into 0.5-1 cm parasagittal slices. Samples for histological analysis were extracted from the periphery and centers of each lobe and subsequently dehydrated and embedded in paraffin. The present investigation was undertaken on a tissue macroarray of seven samples selected from superior lobes of well-characterized COVID-19 patients (patient nos. 1 to 7 of the cohort of Menter et al. [18], 2 females and 5 males, age 68-96 years; Table 1). The tissue macroarray was constructed by dissecting equilateral triangular fragments of 0.5 cm (i.e., 0.108 cm^2^) from paraffin-embedded lung tissue containing typical sequelae of COVID-19 and transferring these fragments to a recipient block, in a manner analogous to that described by Battifora [46]. Tissue microarray cores with 1 mm diameter from age-matched patients, who died of pneumonia and ARDS due to other causes than COVID-19, were used for quantitative evaluation of citH3+ NETs and neutrophils. These reference specimens included 5 cases of bacterial (*Streptococcus pneumoniae*) pneumonia [47] and 5 cases of non-COVID-19-related DAD [48], as well as 5 reference control lungs without pathology.

Immunohistochemistry was performed for the neutrophilic enzyme MPO, the chromatin decondensation marker citH3, the carcinoembryonic antigen-related cell adhesion molecule CD66b, and the glycan determinant CD15/Lewis x, a distinguishing marker of human myeloid cells, the latter two reported as highly expressed in low-density neutrophils (LDNs) [49]. The following primary antibodies were used: polyclonal rabbit-anti-MPO (Ref. 760-2659; Ventana-Roche, Rotkreuz, Switzerland), polyclonal rabbit anti-histone H3/citrulline R2+R8+R17 (ab5103; Abcam, Cambridge, UK), rabbit polyclonal anti-CD66b (ab197678; Abcam, Cambridge, UK), and monoclonal anti-CD15 (clone MMA, Ref. 760-2504; Ventana-Roche, Rotkreuz, Switzerland). An OptiView DAB IHC Detection Kit in a Ventana Benchmark Ultra autostainer (with MPO and CD15) and AP-conjugated goat anti-rabbit IgG (ab97048, Abcam, UK; with citH3 and CD66b) were used for secondary visualization. Histochemical Prussian-blue staining (Perls' stain) was used to identify macrophages with endogenous iron (hemosiderin) deposits. Hematoxylin (with MPO and CD15), Feulgen-Rossenbeck reaction (with citH3 and CD66b), and nuclear fast red (with Prussian blue) were used as nuclear/DNA counterstains.

Quantification of citH3+ NETs and citH3+ cells with still intact nuclei (the latter being assumed to be neutrophils induced to NETosis) was performed by stereological point counting using ImageJ software. Random nonoverlapping microscopic fields (final size 565 × 433 *μ*m = 244.65 mm^2^) were taken from the citH3-Feulgen double-stained macroarray specimens (5 fields per specimen) and from the similarly stained reference specimens (see above). Microscopic fields were digitally overlaid with a regular 10 × 10 *μ*m square array test system; array crosspoints falling on the target structures were counted and used to calculate area fractions. A nonparametric Kruskal-Wallis test was used to evaluate intergroup differences.

### 2.1. Ethical Approval

The work was conducted in cooperation of both institutions and was approved by the Ethics Committee of Northwestern and Central Switzerland (Ethikkommission Nordwest- und Zentralschweiz), Project-ID 2020-00969, decision of May 19^th^, 2020 (formal letter in German).

## 3. Results

Immunohistochemical analyses for all NETosis markers produced variable results between tissue samples of different patients and demonstrated that signals were not restricted to the producer cells but also present at sites of secondary dissemination.

**Figure 1:**
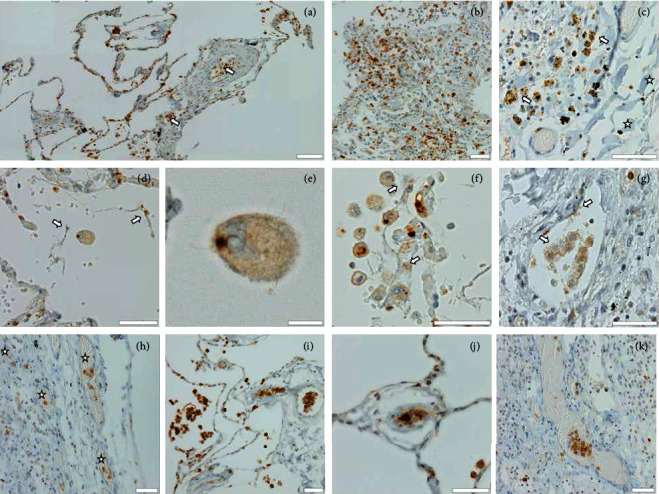
Immunostaining for myeloperoxidase (MPO) (brown), DNA/nuclei counterstained with hematoxylin (blue). (a) Overview image showing moderate alveolar septal infiltration by MPO+ cells, mostly of neutrophilic origin; prominent clustering also in capillaries and medium-sized blood vessels (arrows). (b) Accumulation of MPO+ cells in a bronchiolized alveolus. (c) Cluster of enlarged MPO+ cells with reticulate and granular inclusions (arrows) in DAD, next to deposits of hyaline membrane (asterisks). (d) Enlarged ovally shaped cell with reticulate MPO+ filling in an alveolar space; adjacent fibrous structures with attached MPO+ material (arrows) stain for DNA and are thus considered as NETs. (e) Higher magnification of the cell in (d): the reticulate filling is partly associated to DNA and frays out at the periphery, thus being also considered as NETs. (f) MPO+ cells similar to those in (c–e) and patches of free NETs (arrows) at a damaged alveolar septum. (g) Aggregations of NET-forming cells and NETs in a blood vessel; note partial overlay of the MPO stain and the DNA counterstain (arrows). (h) MPO+ cells in microthrombotic capillary occlusions (asterisks). (i–k) Microthrombotic formations that consist entirely (i, j) or largely (h) of MPO+ cells ((h) in DAD tissue). Scale bars: (a) 100 *μ*m, (e) 10 *μ*m, and (b–d) and (f–k) 50 *μ*m.

Immunostaining for MPO demonstrated an extensive neutrophil infiltration mainly in the interalveolar septal space. With some variation in incidence between samples, scattered MPO+ cells were detected on alveolar surfaces and alveolar lumens and clumped and/or arranged along the inner lining of the capillary endothelium and other small vessels (Figure 1(a)). Large numbers of MPO+ cellular aggregates were found in and associated with areas of proliferative diffuse alveolar damage (DAD) in particular (Figures 1(b) and 1(k)). Ample deposits of hyaline membrane (asterisks in Figure 1(c)) were usually less densely populated. In some areas, MPO+ cells appeared spread out, with large oval cell bodies and speckled and/or reticulate inclusions partly costaining for DNA (arrow in Figure 1(d), detail in Figure 1(e)). In focal and/or patchy patterns, MPO-stained particles were also observable at extracellular sites. MPO signals were found at various sites in the parenchyma in fine granular deposits or fibrous meshworks, frequently associated with alveolar surfaces and inner linings of alveolar septa and capillaries, displaying an eroded appearance (Figures 1(f) and 1(i)), and cemented between MPO+ cells in microthrombi (Figures 1(g)–1(k)). From previous investigations [50, 51], we postulate that these formations are the histomorphological correlate of NETs. Similar to MPO+ cells, deposits in hyaline membranes were hardly interspersed with MPO+ fibrous matter.

**Figure 2:**
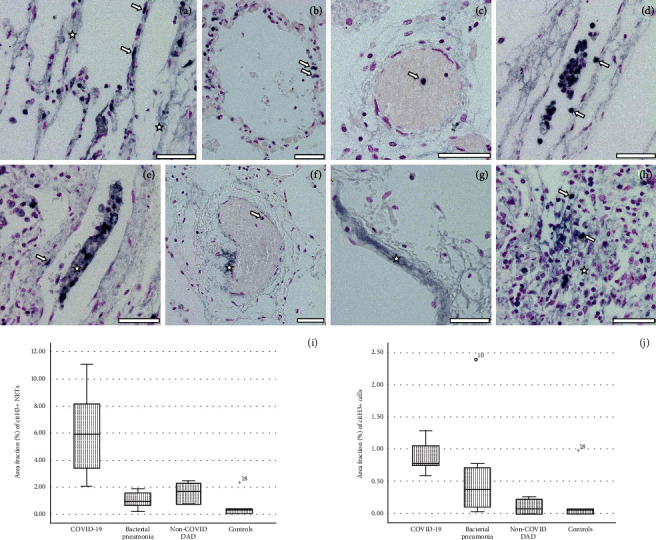
Immunostaining for citrullinated histone H3 (citH3) (dark blue), DNA/nuclei counterstained with Feulgen method (purple). (a, b) citH3+ cells (most likely NET-forming neutrophils) reside abundantly in alveolar walls (arrows); NET-like DNA meshwork formation along a damaged alveolar wall (asterisks). (c) citH3+ neutrophil in a microthrombus. (d, e) citH3+ neutrophils attached to, and inserted into, vascular endothelia (arrows); microthrombi in the lumina consist largely of citH3+ neutrophils clotted together by reticulate DNA (asterisk in (e)). (f) Accretion of NETs (asterisk) on the outer face of an occluded vessel with a microthrombus containing a citH3+ cell (arrow). (g) NETs (asterisk) in an eroded alveolar wall. (h) Congregation of citH3+ neutrophils (arrows) and NETs (asterisk) in proliferative DAD. Scale bars: 50 *μ*m. (i, j) Box-and-whisker plots showing interquartile ranges and medians of area fractions (%) occupied by citH3+ NETs (i) and citH3+ neutrophils (j) in citH3/Feulgen double-stained COVID-19 lung tissue and in similarly stained reference specimens of non-COVID-19 bacterial pneumonia and DAD and pathologically normal control lungs.

Staining patterns for citH3 were broadly in accordance with those for MPO (and also with those described for CD66b below). Single or clustered cells at various locations throughout the lungs stained positive for citH3, indicating an initiation of PAD4-mediated chromatin decondensation. Similarly to MPO, the most commonly positively stained sites included alveolar septa (Figures 2(a) and 2(b)), blood vessel walls (Figures 2(c)–2(e)), and thrombotic occlusions (Figures 2(c)–2(f)). Some thrombi were almost entirely composed of citH3+ cells (Figures 2(d) and 2(e)). In agreement with the MPO and CD66b staining patterns, citH3 was frequently also found in fine granular and/or fibrous extracellular structures and often associated with the DNA stain, thus corroborating our assumption that these structures represent NETs (asterisks in Figures 2(f)–2(h)). As with MPO, NETosis demonstrated a patch-like distribution, additionally found inside of and interspersed with the constituents of thrombi (asterisk in Figure 2(e)). A particularly high presence of intra- and extracellular citH3 was similarly seen in proliferative DAD (Figure 2(h)).

Results of the quantification of citH3+ NETs and neutrophils indicated a clearly higher prevalence of both target variables in COVID-19 lungs as compared to *Pneumococcus* pneumonia and non-COVID-19 DAD (p4, lines 139-144, and Figures 2(i) and 2(j)). Differences are statistically significant between COVID-19 and bacterial pneumonia for citH3+ NETs, between COVID-19 and non-COVID-19 DAD for citH3+ cells with still intact nuclei, and between COVID-19 and control lungs devoid of pathology for both target variables (*p* < 0.05 each).

**Figure 3:**
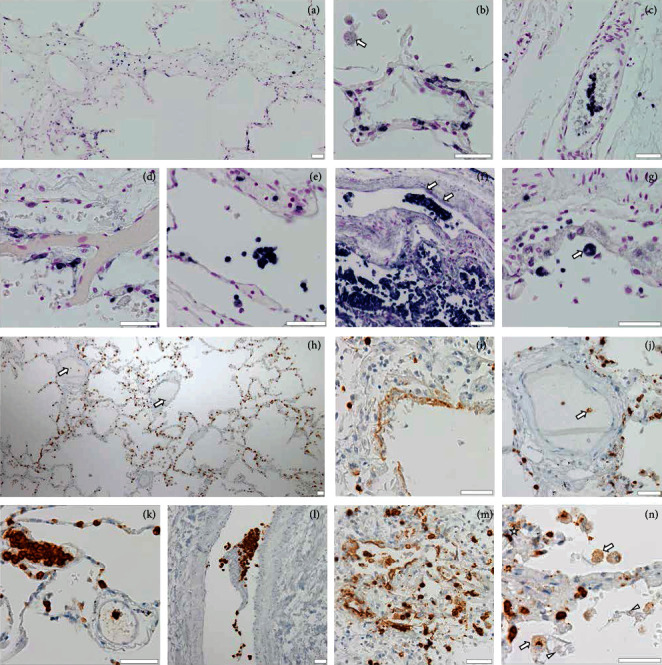
Immunostaining for the low-density neutrophilic (LDN) markers CD66b (dark blue (a–g)) and CD15 (brown (h–n)), DNA/nuclei counterstained with Feulgen method (purple (a–g)) and hematoxylin (blue (h–n)). (a) Overview image showing the distribution of CD66b+ cells; note agglomerations in alveolar septa. (b) Alveolar septal infiltration by CD66b+ cells (most of them presumably LDNs); enlarged oval cells with reticulate CD66+ fillings also staining for DNA in the alveolar space at top left (arrow). (c) Microthrombus consisting of CD66b+ presumed LDNs and NETs. (d) NET-forming CD66b+ cells next to a clotted capillary bifurcation. (e) Clustering of CD66b+ cells in alveolar spaces. (f) Extensive accumulation and microthrombus formation by CD66b+ cells in DAD; spots of NETs in a blood vessel wall (arrows). (g) Large CD66b+ cell (presumably macrophage) in an alveolar space. (h) Overview image showing the accumulation of CD15+ cells in alveolar septa and the occurrence of such cells in vascular clots (arrows). (i) CD15+ NET-like extracellular material interstratifying an eroded alveolar septum. (j) NET-forming CD15+ cell (arrow) in an arteriolar microthrombus. (k) Top left: microthrombus exclusively formed by CD15+ cells (presumably LDNs); bottom right: “conventional type” microthrombus mainly consisting of anucleated material but interspersed with NETs surrounding a strong CD15+ cell. (l) Massive mural microthrombotic accretion of CD15+ cells in a medium-sized arteriolar vessel with tangentially sectioned wall. (m) CD15+ cells and NETs in proliferative DAD. (n) Alveolar spaces with enlarged ovally shaped CD15+ NET-forming cells (arrows) similar to those shown for MPO and citH3; nearby NETs costain for DNA and CD15 (arrowheads); NETs also abundantly intersperse wall tissues (asterisk). Scale bars: 50 *μ*m.

Large amounts of cells in all samples stained positively for CD66b and CD15. Many cells intensely expressing these granulocyte markers likely represent LDNs that are strongly prone to NETosis [49]. Localization and distribution of CD66b+ and CD15+ cells mirrored the patterns described for MPO+ cells, being particularly present in alveolar walls and spaces (Figures 3(a), 3(b), 3(e), 3(h), and 3(k)), in and around blood vessels (Figures 3(c), 3(d), 3(f), 3(j), 3(k), and 3(l)), in NET-associated thrombi (Figures 3(c), 3(f), and 3(l)), and occasionally in alveolar spaces (Figure 3(e)). The largest conglomeration of CD66+ cells was also here found in samples of proliferative DAD (Figures 3(f) and 3(m)). Extracellular staining for CD66b and CD15 was abundant, with patterns largely conforming to those of MPO, i.e., showing diffuse or fibrous inclusions in blood vessels and alveolar walls (arrows in Figures 3(f), 3(i), and 3(j)) and roundish patches of speckled or reticulate matter (Figures 3(g) and 3(n)) comparable to the diffuse remnants of MPO+ neutrophils (cf. Figures 1(d) and 1(e)). Some blood clots consisted almost exclusively of strongly CD66b+ or CD15+ cells (i.e., presumed LDNs), partly intermingled with extracellular substance similarly exhibiting positivity for one of these markers (Figures 3(f) and 3(l)).

**Figure 4:**
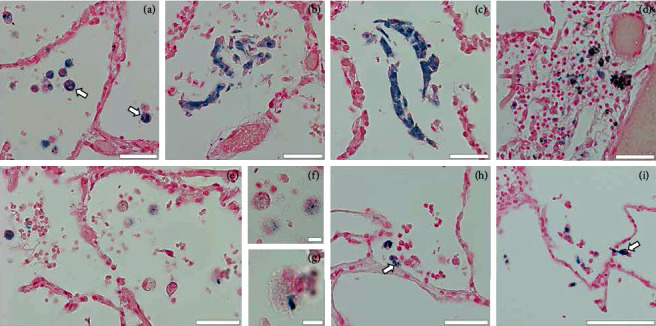
Prussian blue (PB) stain, DNA/nuclei counterstained with nuclear fast red. (a) Individual large cells (arrows) with spotted PB-stained cytoplasmic inclusions and lobulated nuclei in alveoli. (b, c) Mainly chain-like aggregations of PB-stained cells and extracellular substance in alveoli (vascular blood clots all unstained). (d) PB+ cells and extracellular granular deposits scattered among eroded perivascular tissue and hyaline membrane fragments in DAD. (e–g) Roundish patches with reticular fillings and granular PB+ inclusions in alveoli; details in (e, g) illustrate close resemblance to enlarged NET-forming cells in Figures 1–3 (h, i). Smaller PB+ cells in alveoli, one next to a cluster of similarly stained granular deposits (arrow in (h)), another fixed while transmigrating through an alveolar wall (arrow in (i)). Scale bars: (a–e) and (h, i): 50 *μ*m, (f, g): 10 *μ*m.

Six of the seven samples additionally contained relevant numbers of cells of different sizes that stained with Prussian blue (Perls' stain). Large Prussian blue-stained cells mainly formed loose groups or clusters in alveoli (Figures 4(a)–4(c)), but single cells also were detected in interalveolar spaces and adjacent to blood vessels (Figure 4(d)). Prussian blue-stained granules were also observed in roundish patches that bore resemblance to outspread neutrophils (Figures 4(e)–4(g); compare Figures 1(d) and 1(e) and Figures 3(j) and 3(k)) and in other (smaller) cells of varying locations (Figures 4(h) and 4(i)). Such granules were also found intermingled with extracellular substances, probably including areas of released NETs (Figure 4(g)).

## 4. Discussion

In accordance with previous analyses of COVID-19 lungs (e.g., [40]), immunohistochemistry revealed neutrophilic infiltration of virtually all lung tissue compartments accentuated in interalveolar septa (Figures 1(a) and 1(f), Figures 2(a) and 2(b), and Figures 3(a) and 3(b)) and the inner linings of capillaries (Figures 1(g), 1(i), and 1(k); Figure 2(c); and Figure 3(c)), as well as an accumulation in proliferative DAD in particular (Figures 1(b) and 1(k); Figure 2(h); and Figures 3(f) and 3(m)). The relevance of the latter might not only be seen in a detrimental inflammatory context but also in the light of findings in ARDS of other etiologies, such as acid-induced lung injury, indicating that neutrophils promote alveolar epithelial regeneration via enhancement of type II pneumocyte proliferation [52]. Current evidence suggests that the negative effects of NETs will far outweigh any possible benefit. Indeed, immunostaining for MPO, citH3, CD66b, and CD15 unambiguously demonstrates an abundant presence of NETs and NET-generating neutrophils at sites of alveolar damage (e.g., Figure 1(a); Figures 2(c) and 2(g); and Figures 3(b), 3(i), and 3(n)) and intravascular clotting (Figures 1(h), 1(i), and 1(k); Figures 2(c), 2(e), and 2(f); and Figures 3(c), 3(j), and 3(k)) in COVID-19 lungs. The thereby observed ovally shaped cells with reticulate inclusions of MPO+, CD66+, or CD15+ DNA (Figures 1(d) and 1(e) and Figures 3(g) and 3(n)) bear a striking resemblance with NET-shedding neutrophils known from our *in vitro* induction studies (A.O., unpublished work, Fig. S1 in Supplementary materials). A state of detrimentally enhanced NETosis seems to be also mirrored in the serological data before death (Table 1), particularly by the high levels of CRP and lactate dehydrogenase (LDH) (role in NETosis addressed by [53]), and strong neutrophilia in four of the seven patients.

Together, this clearly substantiates our initial hypothesis that COVID-19-associated DAD and DAD in general inherently involve and are likely aggravated by excessive NETosis. This is, in principle, similar to what has been shown for influenza-related DAD [23, 54] and for bacterial pneumonia [32, 55]. However, the present morphological findings together with the results of the comparative quantitative analysis of citH3 (Figures 2(i) and 2(j)) point toward potentially more drastic adverse effects of NETosis in COVID-19.

Implications of the present results for COVID-19 thromboinflammatory pathology seem to be multifaceted. Our current observations provide important further support of previous findings in COVID-19 patients revealing DAD with particular signs of vascular dysfunction in lungs and other organs [13, 16–18]. Validation that the microvascular thromboses seen are not simply cadaveric clots (*cruor sanguinis*) but true microthrombi comes from the finding that these formations contain well-formed, intensively immunopositive fibrin casts of 5-20 *μ*m in dimension [18] and from broad clinical evidence of thrombotic microangiopathy even in nonlethal COVID-19 [56]. Although microthrombosis is not an exclusive feature of COVID-19 but a potential complication of DAD in general, results from our research indicate a much higher prevalence of such microthrombi in COVID-19. A previous study on the same cohort found a ninefold increase of alveolar capillary microthrombi per standard area of injured lung tissue in the same 7 COVID-19 patients compared to influenza patients [17]. Together with several lines of evidence from the literature (e.g., summarized by [12]), this supports the conclusion that COVID-19 is associated with a more specific form of DAD characterized by extreme hypercoagulability.

Our current study provides evidence that some microthrombi in COVID-19 consist almost entirely of citH3+, CD66b+, and CD15+ cells (Figures 2(d) and 2(e) and Figures 3(e), 3(f), 3(k), and 3(l)) and points to an instrumental role of strongly NETosis-prone LDNs in COVID-19 vascular clotting. The finding confirms and further specifies related results of Jiménez-Alcázar et al. [57], Middleton et al. [41], and Leppkes et al. [40]. Evidence from cancer research suggests that LDNs represent a mixture of immature and mature variants in proportions contextually varying with diseases [58, 59]. It has been proposed that immature LDNs are in fact a class of myeloid-derived suppressor cells able to interfere with T cell*-*mediated immune responses, while mature LDNs are converted from normal density neutrophils (NDNs) by a mechanism depending on host and/or pathogen-derived factors, thereby similarly acquiring immunosuppressive qualities [58, 60]. As shown in tuberculosis, LDN-derived NETs may induce a vicious cycle by means of increased NDN-to-LDN conversion [61]. Recent synoptic analysis suggests a considerable pathophysiological role of human LDNs in a plethora of conditions, such as acute and chronic infections, inflammation, cancer, and pregnancy [62]. The role of LDN-derived NETs in COVID-19 thus warrants further investigation.

Large Prussian blue-stained cells (Figures 4(a)–4(c) and 4(e)) are in all probability hemosiderin-laden macrophages. Their presence is consistent with findings from analysis of bronchoalveolar lavages (BAL) from COVID-19 patients [63]. Together with the presence of hemosiderin granules in presumably NET-forming neutrophils (Figures 4(e)–4(g)) and other mobile cells (Figure 4(i)), and at various extracellular sites, this may prompt considerations about the scope of SARS-CoV-2-induced damage on erythrocyte hemoglobin. Indeed, alveolar macrophages (and likely other phagocytes) collecting free iron ions derive from iron displacement from the heme porphyrin [63–65]. As a caveat, clustered large Prussian blue+ cells bear remarkable similarity to giant (pseudo-)syncytia also occurring in COVID-19 lungs. These were recently classified as of pneumocyte origin due to their staining for surfactant-A, thyroid transcription factor 1, and napsin A [66]. Relations with iron ion uptake remain to be determined.

### 4.1. Note on Tissue Sampling

Careful inspection of the chosen COVID-19 tissue samples together with the use of a macroarray of comparably large fragments as opposed to conventional tissue microarray cores (usually 1 mm in diameter, i.e., 0.785 mm^2^) as well as punching control tissues in triplicates served to prevent confounding of the obtained results due to tissue microheterogeneity.

## 5. Conclusion

Overall, our immunomorphological findings confirm and considerably expand upon the recent histopathological findings on NETosis in COVID-19 lungs. NETs are abundantly present in respective seriously damaged respiratory tissue and thrombotic vascular occlusions, unbiased by formalin autofluorescence. Our results also clearly support a relevant contribution of LDNs to COVID-19 pathophysiology, with an emphasis on vascular blockage and microthrombus formation. Further elucidation of the underlying mechanisms allows for a more diversified understanding of neutrophil heterogeneity and effects of COVID-19 immune dysregulation.

## Figures and Tables

**Table 1:** Patient-related data.

Parameter	Value
General	
Sex: male/female ratio	5 : 2
Age (years), mean (range)	78 (66–96)
Hospitalization time, mean (range)	5.1 (3–9)
Cases with ICU admission (%)	2 (29)

Comorbidities, *n* (%)	
Hypertension	7 (100)
Coronary artery disease	5 (71)
Smoker	4 (57)
BMI (kg/m^2^) (range)	30 (23-44)
Overweight/obesity (WHO grade 1/2/3)^∗^	4/0/1/1 (57/0/14/14)
Diabetes mellitus, type 2	3 (43)
Chronic obstructive pulmonary disease (COPD)	1 (14)
Malignancy	2 (29)

Initial clinical presentation, *n* (%)	
Cough	5 (71)
Fever	4 (57)
Dyspnea/tachypnea	3 (43)
Diarrhea	1 (14)
Acute or acute-on-chronic kidney injury	2 (29)

Last laboratory findings before exitus	
CRP (mg/l) (range)	262.4 (82.1–512.3)
LDH (<135 U/l) (range)	605.1 (236–1605)
Hemoglobin (120-180 g/l) (range)	108.3 (73–132)
Anemia, *n* (%)	6/7 (86)
Total white blood cell count (3.5 − 10.0 × 10^–9^/l) (range)	10.8 (1.75–27.08)
Neutrophilic granulocytes (2.0 − 8.0 × 10^–9^/l) (range)	9.6 (2.86–25.32)
Lymphocytes (1.0 − 4.0 × 10^–9^/l) (range)	0.6 (0.24–0.96)
Lymphopenia, *n* (%)	7 (100)
Neutrophilia, *n* (%)	4 (57)
Platelets (150–450 × 10^–9^/l) (range)	222.9 (16–400)

^∗^Overweight: BMI 25-29.9; obesity grade I: 30-34.9, grade II: 35-39.9, and grade III: >40.

## Data Availability

The authors confirm that the data supporting the findings of this study are available within the article and its supplementary materials.

## Supplementary Materials

Supplementary Materials As a supplement, we provide a panel of three as yet unpublished images (Figs. S1A-C) from scanning and transmission electron microscopy (SEM and TEM, respectively) taken in the course of our previous research employing *in vitro* induction of NETs [51]. The images document that human neutrophils releasing NETs after stimulation with phorbol myristate acetate (PMA) bear a close morphological resemblance to the large oval-shaped cells filled with reticulate matter staining for NETosis-related marker proteins and DNA that we show in Figures 1(d) and 1(e) and Figures 3(g) and 3(n). The SEM images (Figs. S1A,B) show an irregularly rounded patch of NETs encircling the still compact remains of its cell-of-origin (S1A), and the remains of a neutrophil in a further advanced stage of NETosis, with thin NET strands projecting radially outward and being bundled into a tail-like extension (S1B). The TEM image (Fig. S1C) was taken from an 80 nm horizontal section through a cell similar to that in Fig. S1A. The cell shows a largely intact outer membrane and a still recognisable nucleus with a partly preserved nuclear membrane. Decondensed chromatin is present in the cytoplasm. Threads of NETs decorated with vesicles attach to and project from the cell's surface.
