# Supplementary material for: Neutrophil Extracellular Traps in Fatal COVID-19-Associated Lung Injury
Source: Dis Markers. 2021 Jul 30;2021:5566826. doi: 10.1155/2021/5566826 (PMC8337148; doi:10.1155/2021/5566826)
Supplement: Supplementary Materials — As a supplement, we provide a panel of three as yet unpublished images (Figs. S1A-C) from scanning and transmission electron microscopy (SEM and TEM, respectively) taken in the course of our previous research employing in vitro induction of NETs [51]. The images document that human neutrophils releasing NETs after stimulation with phorbol myristate acetate (PMA) bear a close morphological resemblance to the large oval-shaped cells filled with reticulate matter staining for NETosis-related marker proteins and DNA that we show in Figures 1(d) and 1(e) and Figures 3(g) and 3(n). The SEM images (Figs. S1A,B) show an irregularly rounded patch of NETs encircling the still compact remains of its cell-of-origin (S1A), and the remains of a neutrophil in a further advanced stage of NETosis, with thin NET strands projecting radially outward and being bundled into a tail-like extension (S1B). The TEM image (Fig. S1C) was taken from an 80 nm horizontal section through a cell similar to that in Fig. S1A. The cell shows a largely intact outer membrane and a still recognisable nucleus with a partly preserved nuclear membrane. Decondensed chromatin is present in the cytoplasm. Threads of NETs decorated with vesicles attach to and project from the cell's surface. [file 5566826.f1.zip › 5566826.f1.docx]

**Supplement**

**Fig. S1.** Scanning electron microscopy images (A, B) and transmission electron micrograph (C) of NET release by human neutrophils after in vitro NETosis induction with phorbol myristate acetate (PMA). (A) Irregularly rounded patch of released NETs encircling the still compact remains of its cell-of-origin. (B) More fragmented remains of a neutrophil in a further advanced stage of NET release. Thin NET strands project outward radially and are bundled into a tail-like extension. (C) 80 nm horizontal section through a cell similar to that in A. The cell shows a largely intact outer membrane, a still recognisable nucleus with a partly preserved nuclear membrane, and discharged decondensed chromatin in the cytoplasm. NET threads decorated with vesicles attach to the outer surface and project therefrom.
